# Molecular Research in Cardiovascular Disease

**DOI:** 10.3390/ijms22137199

**Published:** 2021-07-04

**Authors:** Maria Dorobantu, Maya Simionescu, Nicoleta-Monica Popa-Fotea

**Affiliations:** 1 Department 4-Cardiothoracic Pathology, University of Medicine and Pharmacy Carol Davila, 8, Eroii Sanitari Bvd., 050474 Bucharest, Romania; fotea.nicoleta@yahoo.com; 2 Department of Cardiology, Clinical Emergency Hospital of Bucharest, 8, Calea Floreasca, 014461 Bucharest, Romania; 3 Institute of Cellular Biology and Pathology “N. Simionescu” 8, B.P. Hasdeu Street, P.O. Box 35-14, 050568 Bucharest, Romania; maya.simionescu@icbp.ro; 4 Romanian Academy, 125, Calea Victoriei, 010071 Bucharest, Romania

Cardiovascular diseases have attracted our full attention not only because they are the main cause of mortality and morbidity in many countries but also because the therapy for and cure of these maladies are among the major challenges of the medicine in the 21st century. We designed this Special Issue with the belief that biomedical research will pave the road which in the near future will lead us to find remedies and cures for the cardiovascular diseases.

The collected contributions of this Special Issue of International Journal of Molecular Sciences, “*Molecular Research in Cardiovascular Disease*”, exposes various mechanisms involved in the atherosclerotic process and, furthermore, in therapies implicated in the myocardial recovery post various cardiac diseases, mainly myocardial infarction (MI).

A short consideration of the presented papers could start with Popescu et al. [1], who show in their article that mesenchymal stromal cells (MSC) in conjunction with endothelial colony forming cells (ECFC) augment the therapeutic effect of MSC and enhance the angiogenic properties of ECFC. The use of these types of cells together in a mouse model of MI resulted in a better recovery postinfarction as assessed by left ventricular ejection fraction (LVEF) and stroke volume.

Schumacher et al. showed in their study that miRNA-155 upregulation after MI induces cardiac remodeling through inflammation, fibroblast recruitment and cardiomyocytes inflammation. Although genetic depletion of miR155 would be expected to reduce the area of infarction and improve LVEF, in a dyslipidemic mouse model of MI, the deletion of miR155 did not improve cardiac function, only reduced myofibroblasts’ density in the postinfarction scar [2], with no favorable impact on the reduction of fibrosis and/or increase in angiogenesis. Further studies are needed to pinpoint the mechanisms through which miR155 influences the phenotype and viability of myofibroblasts under various spatial and temporal circumstances.

As reported, miRNAs have emerged as promising biomarkers for diagnosis and prognosis, and more recently as potential therapeutics, as these are involved in the development of ischemic heart disease (IHD) at all levels (atherogenesis, angiogenesis, inflammation, platelet activation and aggregation, lipid metabolism). As comprehensively synthetized by Scarlatescu et al. [3], certain miRNAs play a favorable role in the reduction of mortality and left ventricular remodeling (miR-150, 145, 101), while many others have negative outcomes in MI (miR-27a, miR155, miR-1, miR-24, etc.). As shown by Scarlatescu et al., as many as 213 studies have been published regarding miRNAs, but only a few of them are in vivo studies with relevant impact. Bejerano et al. show that boosting the expression of miR-21 in the macrophages attracted to the infarction area in the first days promotes the acceleration towards increased angiogenesis, a reduction in apoptotic cells and attenuation in left ventricle remodeling after MI [4]. In addition, Dong et al. report that the upregulation of miR-21 after preconditioning reduces cell apoptosis and the area of infarction by almost a third [5].

Dysregulated expression of arterial miRNAs was found also in treatment-naïve patients with giant cell arteritis (GCA) as shown by Kuret et al. [6]. The following miRNAs were proved to be new deregulated molecular factors in GCA temporal artery biopsies: MiR-424-3p, -503-5p, KLF4, PELI1 and YAP1. Correlation-based analysis of miRNAs and mRNA identified KLF4 as a candidate target gene of deregulated miRNAs in GCA. However, there was no correlation between mRNA or miRNAs, symptoms and severity of GCA, with further studies being needed for a comprehensive understanding of the physiopathology of GCA.

Familial hypercholesterolemia (FC) represents one important risk factor for IHD; apart from lipid accumulation within the intima, another mechanism often encountered in FC is inflammation. By using a particular system biology, Garcia-Arguinzonis et al. [7] investigated the role complement C3 plays in atherosclerosis on the phenotype and function of human lipid-loaded vascular smooth muscle cells (VSMCs). By spectrometry and differential proteomics, it was established that complement C3 was more abundantly expressed in atherosclerotic–extracellular matrix, compared with normal segments. Even in subjects with sub-clinical atherosclerosis, circulating C3 levels were higher, and in cell cultures the expression of C3, C3aR and C3b/iC3b (active fragments) was augmented. All these data suggest that the complement C3 system is involved in the progression of atherosclerosis by vascular remodeling. Interestingly, in subjects with FC there was significant activation of the innate immune system, with local complement C3 accumulation in the atherosclerotic ECM of the aorta, while the circulating complement C3 levels did not seem to be a sensitive measure of the plaque burden severity, with this finding underlying that the main source of complement C3 remains the vascular resident cells. The same study shows that the inhibitory effect of aggregated LDL on VSMCs’ migration is ameliorated by the presence of exogenous complement C3a to a level that did not significantly differ from the migration capacity of the control group, reflecting the novel role of complement C3 apart from its well-known function in inflammation and immunity.

Communication between cells is essential for the normal function of organisms. Extracellular vesicles (EV) are recently discovered cell-derived elements encompassing apoptotic bodies, ectosomes and exosomes. Extracellular vesicles have been shown to act in the development of atherosclerotic plaques, with a negative effect activating the pro-inflammatory cytokines IL-8, IL-1 and IL-6 and enhancing the adhesion properties of the endothelium. Moreover, EV promote vascular calcification and induce mineralization. As reviewed by Georgescu and Simionescu [8], EV can deliver molecules to target cells. Berezin and colleges pinpointed that stem-cell-derived microvesicles inhibited vascular remodeling by transfer of miR-125 and miR-22 and, furthermore, by the enhancement of microvascular endothelial cells [9]. Extracellular vesicles also proved to be useful therapeutics in emerging SARS-CoV2 infections. The administration of mesenchymal stem-cell-derived EV administered intravenously to COVID-19 subjects with moderate to severe pathology downregulated the cytokine storm and restored oxygenation. Other trials with no official results yet, employ the EV as therapeutics in COVID-19, synthetized in the review of Georgescu and Simionescu mentioned above.

Keeping within the same line of research, endothelial EV have a role in endothelial physiology, as exposed by Mathiesen et al. [10]. Injured endothelial cells can be repaired and regenerated by complement-induced apoptosis by shedding EV rich in caspase-3, protecting the endothelium against stress, as Abid Hussein et al. showed [11]. Other reports by Brill et al. highlight that platelet-derived vesicles can induce vascular endothelial growth factor-dependent angiogenesis promoting revascularization in IHD [12]. Apart from their role in normal development and the homeostasis of the endothelium, EV also play a role also in endothelium plasticity and dysfunction, such as angiogenesis, endothelium inflammation, vasoreactivity and thrombosis. Extracellular vesicles proved useful as biomarkers for endothelial damage with prognostic values. Nozaki’s group showed that endothelial-cell EV independently predicted cardiovascular events in patients with a high risk of heart disease [13]. Despite the many studies that highlight the contribution of EV to endothelium dysfunction, their use as therapeutics encounters a lot of challenges, starting from isolation, purification and preparation of EV, balancing the equilibrium between harmful and beneficial effects of EV populations. Mathiesen et al. conclude in their review that personalized medicine should not refer only to holistic treatment, but also to personalized healing of the endothelial cell, that altogether contribute to the healing of the most prevalent killer of our century, cardiovascular disease.

Lupu et al. [14] described in 2011 the androgen-dependent Tissue Factor Pathway Inhibitor (TFPI)-Regulating Protein (ADTRP), the major inhibition of TF-dependent pathway of coagulation of endothelial cells. Many single polymorphisms correlated with the risk of cardiovascular diseases, deep-venous thrombosis or thromboembolism have been reported since then. The same research group, led by Lupu, discovered the critical role of ADTRP in vascular development and vessel integrity. Single nucleotide polymorphism- rs6903956- in ADTRP [15] was more prevalent in IHD, but the exact mechanisms that link low ADTRP expression to the increased risk of IHD are still under research. Nevertheless, Ooi and colleagues advanced the idea that every 100 pg/mL increase in ADTRP decreases the risk of IHD by 9% [16]. ADTRP is a transmembrane protein with its location in lipid rafts/caveolae, with no potential release from the cells; it can be speculated that a circulating pool of ADTRP may reflect its release, but to demonstrate this, further experimental data are needed. For the moment, in depth studies to investigate the function of ADTRP are needed to understand how it functions and its involvement in diseases.

Regulatory light chains (RLCs) have an important function in cardiogenesis, being the first markers expressed in the primitive heart tubes of vertebrates. In heart failure, there is a reduction in RLC phosphorylation levels, and restoring RLC phosphorylation generates an increase in cardiac muscle contractility. Interestingly, RLC phosphorylation can rescue the pathological effects arising from mutated RLCs. In the review of Markandran and colleges [17], it is shown that pseudophosphorylation of D166V-mutated RLC prevents fibrosis [18], and phosphorylation of R58Q- and A13T-mutated RLC restores calcium binding to RLC [19]. Altogether phosphorylated RLCs have a potential role in rescuing the progression of heart diseases. Biochemical protein exchange experiments are of uppermost importance in RLCs because muscle cell contractile function can be monitored before and after the exchange; unfortunately, the RLC exchange can be made only in isolated solutions as the protein does not diffuse into the membrane. All these are good premises for the near-future introduction of RLC-based treatments.

The contributions published within the Special Issue “*Molecular Research Cardiovascular Disease*” are excellent examples of the advances made in the study of the intimate mechanisms of cardiac diseases. We would like to thank all the eminent contributors and the reviewers for their efforts to provide great, up-to date, interesting articles, and also to the excellent editor, Shanny Li, for helping throughout our endeavor to provide a modern and interesting Special Issue of the International Journal of Molecular Sciences.

## Data Availability

Not applicable.
